# The dynamic association between physical exercise and depressive symptoms in China: an age-period-cohort analysis using the HAPC-CCREM and the moderating role of urban–rural disparities

**DOI:** 10.3389/fpubh.2026.1869282

**Published:** 2026-07-03

**Authors:** Fuxiang Yu, Long Niu, Boyang He, Ningyi Zhang, Ce Guo

**Affiliations:** 1 Business School, Hangzhou City University, Hangzhou, China; 2 School of Physical Education and Sport, Xi’an Jiaotong University, Xi’an, China; 3 School of Athletic Performance, Shanghai University of Sport, Shanghai, China

**Keywords:** age-period-cohort analysis, depressive symptoms, HAPC model, physical exercise, urban–rural disparities

## Abstract

**Background:**

While the positive association between physical exercise and depressive symptoms (as an indicator of mental health) is well-established, existing research lacks a dynamic life-course and socio-generational perspective. It remains unclear how these associations evolve across different ages, historical periods, and birth cohorts, and how they are moderated by structural factors such as the urban–rural divide.

**Methods:**

Utilizing pooled cross-sectional data from five waves (2010–2017) of the China General Social Survey (CGSS) with 28,618 valid adult samples, this study employs a Hierarchical Age-Period-Cohort Cross-Classified Random Effects Model (HAPC-CCREM) to disentangle age, period, and cohort patterns.

**Results:**

The advantage associated with regular physical exercise in terms of depressive symptoms exhibits a non-linear age pattern suggestive of a U-shape, being strongest among younger and older adults but attenuated during middle age. Cohort analysis reveals that earlier-born cohorts show a stronger positive association between exercise and fewer depressive symptoms. This positive association declined with socio-economic development but shows a resurgence among the post-1990s cohort, suggesting possible links to socio-economic development and shifting values. Urban–rural disparities significantly moderate this association: urban residents exhibit a stronger positive association and more effective stress-alleviating patterns from exercise, whereas the corresponding association for rural residents is comparatively limited.

**Conclusion:**

The positive psychological correlates of physical exercise with respect to depressive symptoms are not static but are dynamically shaped by individual life stages and macro-social transformations. In the context of China’s transition towards post-materialist values, the role of exercise may be shifting from a “luxury enhancer” to a “necessary support.” Future public health strategies should adopt a differentiated approach, considering life-course stages, cohort experiences, and urban–rural structural contexts to implement precise mental health promotion strategies (i.e., reducing depressive symptoms).

## 1 Introduction

Physical exercise is widely recognized as a crucial non-pharmacological strategy for reducing depressive symptoms. The establishment of stable and sustained exercise habits is consistently associated with positive psychological well-being through multifaceted pathways (1). Empirical evidence suggests that consistent physical exercise is associated with a dual correlate for better mental health (i.e., fewer depressive symptoms): physiologically, it relates to the regulation of neuroendocrine systems and neurotransmitter balance, which in turn is associated with fewer symptoms of anxiety and depression (2, 3); socially, it relates to the accumulation of social capital and is associated with stress-alleviating mechanisms within interpersonal networks (4).

A large body of research, including cross-sectional, longitudinal, intervention studies, and meta-analyses, has firmly established the positive association between physical exercise and depressive symptoms. However, the extant literature is predominantly anchored in designs that offer a static snapshot or short-term follow-ups, failing to capture how this association dynamically evolves across the life course and amidst macro-social transformations (5). This limitation is critical because the mechanisms through which exercise correlates with depressive symptoms are deeply embedded within socio-temporal contexts—shaped by individual aging, exposure to specific historical conditions, and shared generational experiences. To decode these complex temporal dynamics, analytical frameworks capable of disentangling Age, Period, and Cohort (APC) patterns are essential (6).

Thus, the novelty of this study lies not in re-establishing the basic exercise-depressive symptoms link, but in three specific contributions that have been largely overlooked in existing literature: (1) Non-linear age pattern – Most previous studies assume a linear or static effect, whereas we hypothesize and test a possibly U-shaped trajectory, exploring when during the life course the advantage associated with exercise may be strongest. (2) Cohort heterogeneity linked to social change – While cross-sectional comparisons cannot separate age from cohort effects, our APC design allows us to examine how the exercise-depressive symptoms association varies across birth cohorts that experienced different socio-historical contexts (e.g., pre-reform, rapid economic growth, post-materialist rise). This is consistent with the view that macro-social transformations may reshape the meaning and utility of health behaviors. (3) Moderating role of structural inequality – We integrate urban–rural disparities (hukou) into the HAPC framework to test whether the psychological returns to exercise differ systematically by structural context, a dimension rarely examined in APC studies.

The research is guided by three core questions: (1) What is the net depressive symptoms advantage associated with maintaining regular exercise habits? (2) How does the psychological correlate of exercise dynamically evolve across an individual’s life cycle (age pattern)? (3) How does the strength of the exercise-depressive symptoms association vary across birth cohorts experiencing different socio-historical contexts (cohort pattern)? Importantly, while we draw on theories of life-course roles, social capital, materialism/post-materialism, and structural inequality to interpret our findings, our empirical model directly tests only age, period, cohort, and hukou heterogeneity. The proposed mechanisms are inferred from the observed patterns and existing literature, but they are not directly measured. Therefore, our interpretations should be considered as plausible explanations rather than empirical evidence for these mechanisms.

To address these questions, this study employs the Hierarchical Age-Period-Cohort Cross-Classified Random Effects Model (HAPC-CCREM) as the primary analytical framework. This model estimates age, period, and cohort effects (as statistical parameters) by treating period and cohort as cross-classified random effects, which provides one strategy to help mitigate the identification problem under specific assumptions about the data structure. It allows for the examination of non-linear age patterns and random slope variations across cohorts. Furthermore, to assess how social structural contexts condition the positive psychological correlates of exercise, key moderators—such as urban–rural household registration (hukou) status—are integrated into the HAPC framework.

Through this approach, the study aims to generate nuanced evidence regarding the dynamic life-course and socio-generational patterns associated with exercise and depressive symptoms. We acknowledge that our repeated cross-sectional design cannot establish individual-level causality; however, by identifying population-level patterns across age, period, and cohorts, we provide novel hypotheses for future longitudinal and intervention studies. The findings are expected to inform differentiated mental health promotion strategies (i.e., reducing depressive symptoms) and contribute to more precise public health strategies.

## 2 Literature review and research hypotheses

Research on the relationship between long-term regular exercise habits and depressive symptoms has been extensively conducted, spanning multiple dimensions including psychology, sociology, education, and sports science (7, 8). While most studies agree that physical exercise positively impacts depressive symptoms, the historical evolution of this relationship has received limited attention. This study analyzes how this relationship evolves over time through three dimensions: age patterns, period patterns, and cohort patterns. Age patterns reflect influences from aging and life course changes; period patterns indicate similar socioeconomic impacts across all demographics during specific historical phases; cohort patterns reveal differences among generations due to early-life experiences and social transformations. Each pattern carries distinct implications, requiring careful differentiation by researchers to accurately estimate trends and reasonably explain mechanisms.

### 2.1 Age patterns in depressive symptoms

A robust body of evidence confirms the positive impact of physical exercise on depressive symptoms, including reduced symptoms of depression and anxiety and enhanced subjective well-being (9, 10). While both acute (short-term) and chronic (long-term) exercise contribute to psychological well-being (11), their functional roles may differ. Acute exercise often serves as an immediate “stress buffer” or “emergency relief” for transient negative emotions, whereas sustained exercise habits function more as a “value-added enhancement,” fostering long-term psychological resilience and maintaining higher baseline levels of mental health (12). Crucially, this relationship is not static across the lifespan but is deeply intertwined with an individual’s life stage and social roles (13). The demands and opportunities associated with different ages—such as career establishment in young adulthood, family and work “role overload” in middle age, and health management in later life—fundamentally shape exercise patterns, motivations, and consequently, their psychological correlates (14).

From an age-graded perspective, exercise behaviors and their psychosocial contexts exhibit systematic variation. Young adults, often characterized by higher energy and a focus on social identity formation, may engage in more intense or socially embedded physical activities (15). For them, exercise can be a critical tool for stress relief from early career pressures and for building social capital (16). Middle-aged individuals, navigating peak career and family responsibilities, often face significant time constraints. Their exercise may shift towards time-efficient or health-maintenance-oriented activities, potentially diluting its role as a primary leisure-based psychological enhancer. For older adults experiencing physical decline, exercise (e.g., walking, Tai Chi) often becomes a central activity for managing health, preserving functional independence, and facilitating social engagement, thereby regaining its salient role for mental well-being (17, 18).

Despite this understanding, a significant gap remains. Most existing studies rely on cross-sectional comparisons or static controls, failing to capture the dynamic, intra-individual evolution of how the positive association between exercise and depressive symptoms waxes and wanes across the life course. This is a critical oversight, as depressive symptoms themselves show life-course patterns. For instance, reports indicate high prevalence of depressive feelings among Chinese adults, with stressors linked to work, marriage, and child-rearing varying in intensity across different life stages. A dynamic perspective is therefore essential to move beyond asking if exercise helps, to understanding when and for whom this positive association is most potent across the aging process.

Based on the above theoretical reasoning and the empirical patterns identified in the full analysis of this study, we formally propose the following hypothesis concerning age patterns:

*H1*: The depressive symptoms advantage associated with regular physical exercise follows a non-linear pattern suggestive of a U-shape across the life course.

### 2.2 Cohort patterns: heterogeneity in the exercise-depressive symptoms association in the context of social change and evolving values

The influence of physical exercise on depressive symptoms is not static but is profoundly shaped by the socio-historical context in which different birth cohorts live. A life course perspective emphasizes that cohorts, defined by their shared birth years and experiences of historical events, develop distinct health behaviors and outcomes (19). The relationship between exercise and psychological well-being in China must be understood against the backdrop of its rapid economic transformation and shifting social values.

Historically, access to and the social meaning of physical exercise have varied dramatically. Prior to the founding of the People’s Republic, organized Physical exercise was largely a privilege of the elite, while the general populace had limited access and awareness (20). Following the establishment of the new state, with the improvement of basic living conditions, mass participation in sports saw a significant increase, becoming part of national building efforts (21). However, the subsequent era of reform, marketization, and intense economic growth introduced new dynamics. As China’s labor market evolved through stages of restriction, liberalization, and intensification (22), the nature and intensity of work-related stress changed across cohorts. For earlier cohorts (e.g., those born before 1949 and the 1950s-60s), individuals who could maintain regular exercise often occupied relatively advantaged socioeconomic positions with lower baseline stress, allowing exercise to function as a “value-added enhancement” for their well-being (23).

The rapid material accumulation and fierce competition characterizing China’s development from the 1980s onwards reconfigured the role of exercise. For middle cohorts (e.g., 70s and 80s births), navigating peak career and family responsibilities under high pressure, personal time became scarce (24). Exercise, often perceived as leisure, could be easily crowded out by work demands. Its positive psychological correlates, while present, might be diluted or less salient amidst overwhelming material pursuits and survival pressures (25). This period reflects a dominance of materialist values, where the immediate returns of economic striving often overshadowed investments in lifestyle-based health maintenance.

A potential shift is observed among more recent cohorts, particularly the post-90s generation. Growing up in an era of greater material abundance, this cohort is increasingly characterized by the emergence of post-materialist values, which prioritize quality of life, self-expression, and psychological well-being over purely economic goals (26). Yet, they face unique, modern pressures such as educational “involution,” career precarity, and digital-era anxieties (27). For them, physical exercise may be undergoing a functional transformation—from a discretionary leisure activity to a strategic, necessary tool for managing stress, building resilience, and asserting control in a high-pressure environment. Consequently, the positive association between exercise and depressive symptoms may show signs of recovery, reflecting its new role as “necessary support” rather than mere “luxury enhancement” (28). This evolution suggests that the cohort pattern is not linear but may follow a trajectory shaped by the interaction between changing macro-social pressures and evolving collective values. Based on this, this study proposes Hypothesis 2.

*H2*: The depressive symptoms advantage associated with regular physical exercise exhibits significant variation across birth cohorts, following a pattern of decline and subsequent recovery.

### 2.3 Urban–rural disparities in the depressive symptoms association with physical exercise

The persistent urban–rural dual structure in China creates fundamental inequalities in the distribution of material resources and the configuration of social environments (29). These structural disparities are posited to significantly moderate the pathways through which health behaviors, such as physical exercise, translate into mental health outcomes (30). Empirical evidence consistently shows that urban residents generally report higher rates of participation in regular physical exercise, which is associated with a stronger positive association compared to their rural counterparts (31). This observed disparity can be theorized through a dual-channel mechanism encompassing both structural and socio-relational dimensions.

First, at the structural-resource level, urban areas concentrate superior public health and recreational infrastructure, including accessible sports facilities, parks, and organized community fitness programs (32). This infrastructure lowers the objective barriers (e.g., availability, convenience, cost) to establishing and maintaining regular exercise routines, making healthy behaviors more feasible for urban dwellers (33).

Second, and potentially more critical, at the socio-relational level, exercise in urban settings is often deeply embedded within richer social contexts. Building on theories of social capital (34) and its specific operation in Chinese society as guanxi (35), regular participation in urban fitness venues (e.g., gyms, sports clubs, public squares) can function as a modern “social capital incubator.” These activities transcend mere physical exertion to become social rituals that facilitate network building, provide sustained emotional support, reinforce social identities, and foster a sense of community belonging. This embeddedness amplifies the positive depressive symptoms correlates of exercise through powerful psychosocial buffering and promotive mechanisms.

Conversely, rural residents face a compounded disadvantage. Macro-structural constraints, such as relative economic precarity and weaker social safety nets (36), limit their capacity to prioritize leisure-time exercise. Furthermore, the physical and social environment in rural areas often lacks the critical mass and institutional settings that facilitate socially embedded exercise (37). Physical exercise for rural residents tends to be more isolated, informal, and less integrated into sustained, supportive social networks. Consequently, while exercise may still offer basic physiological and psychological advantages, the significant “value-added” positive association with depressive symptoms derived from social capital accumulation and stress-buffering social interactions is substantially diminished (38). In this context, exercise may serve more as a basic compensatory coping mechanism rather than a potent promotive factor for enhanced psychological well-being. Based on this, this study proposes Hypothesis 3.

*H3*: The urban and rural living environments play a significant moderating role in the association between individual physical exercise and depressive symptoms.

## 3 Methods

### 3.1 Participants

This study utilizes data from the China General Social Survey (CGSS), a nationally representative, repeated cross-sectional social survey project. The CGSS employs a multi-stage stratified probability sampling design to collect comprehensive information on social structure, quality of life, and health behaviors among Chinese adults aged 17 and above, ensuring high data quality and national representativeness. For the present analysis, we pooled data from five waves conducted in 2010, 2011, 2012, 2015, and 2017. These specific waves were selected based on two primary criteria: (1) the availability of consistent measures for our core variables—mental health and physical exercise habits—across all waves, which is essential for longitudinal trend analysis; and (2) their coverage of a critical period in China’s socio-economic development, allowing us to capture cohort and period patterns amidst rapid social transformation. The initial pooled dataset contained observations from 53,542 respondents.

To construct the analytic sample for the HAPC-CCREM, we applied the following exclusion criteria sequentially: (1) respondents with missing values on the dependent variable (depressive symptoms) or the primary independent variable (physical exercise) were excluded; (2) respondents with missing data on any of the essential control variables (e.g., age, gender, education, household registration, income) were also removed to ensure model estimation integrity. This rigorous data-cleaning process resulted in a final analytic sample of 28,618 valid observations. Comparisons showed no substantial differences between included and excluded respondents. The majority of excluded cases were due to missing data on annual income and physical exercise. Comparisons using t-tests and chi-square tests indicated that excluded respondents were slightly older and more often rural residents, but no significant differences in gender or educational attainment were detected. Therefore, the final sample remained sufficiently representative for the APC analysis. Thus, the final analytic sample remained broadly representative of the Chinese adult population. The demographic composition of the final sample is summarized in Table 1.

**Table 1:** Descriptive results of variables in CGSS 2010–2017.

Variable name	Variable description	Mean	Standard deviation	Min/Max
Dependent variable				
Depressive symptoms	Always depressed = 1; often = 2; sometimes = 3; rarely = 4; never = 5	3.900	0.976	1–5
First layer variables				
Physical exercise	Good habits = 1; no = 0	0.395	0.489	0–1
Sex	Male = 1; female = 0	0.481	0.499	0–1
Hukou	Urban = 1; rural = 0	0.460	0.498	0–1
Educational Attainment	Illiterate = 1; primary school = 2; junior high school = 3; senior high school = 4; university = 5; university or above = 6	2.215	1.279	1–6
Age	continuous variable	44.381	15.193	17–96
Working condition	0 = no work; 1 = agriculture; 2 = non-agricultural	1.135	0.885	0–2
Marital status	0 = unmarried; 1 = married	0.797	0.402	0–1
Quantity of children	Continuous variables from 0 to 11	1.500	1.143	0–11
Log of income	Continuous variables 0–16.11	8.393	0.875	0–16.11
Province	1 = East; 2 = Central; 3 = West	1.795	0.822	1–3
The second layer of variables				
For generations	Ten years is an era	——	——	1949–1999
Period	The period of investigation	——	——	2010–2017

### 3.2 Measures

#### 3.2.1 Dependent variable

Depressive symptoms (as an indicator of mental health). This was assessed using a single item from the CGSS questionnaire: “How often have you felt depressed in the past four weeks?” Responses were recorded on a 5-point Likert scale: 1 = “Always,” 2 = “Often,” 3 = “Sometimes,” 4 = “Rarely,” and 5 = “Never.” A higher score indicates less frequent depressive feelings, thus reflecting better mental health status (i.e., fewer depressive symptoms). We acknowledge that this single item captures only the depression dimension, not the full spectrum of mental health. However, similar single-item measures of depressive symptoms are commonly employed in large-scale social surveys, including the CGSS, due to their brevity and efficiency. Peer-reviewed research using CGSS data has employed this specific item to operationalize and study depression, providing evidence for its utility in the Chinese context (39). Our findings should therefore be interpreted specifically in relation to depressive symptoms.

#### 3.2.2 Independent variable: physical exercise

The questionnaire asked in detail: “Do you engage in physical exercise in your spare time?” The original response options were: 1 = “Every day,” 2 = “Every week,” 3 = “Every month,” 4 = “Once a year or less,” and 5 = “Never.” To distinguish between habitual exercisers and non-habitual exercisers, this variable was dichotomized. Options 1 through 3 were classified as “good exercise habits” (coded as 1), indicating regular exercise. Options 4 and 5 were classified as “poor exercise habits” (coded as 0).

#### 3.2.3 Cohort and period

Cohort and period information was derived from the reported birth year and survey year. Respondents ranged in age from 17 to 96 years at the time of the surveys. For the Age-Period-Cohort (APC) analysis, birth cohorts were grouped into generational intervals: the pre-1949 cohort, and then decadal cohorts (post-1950s, post-1960s, post-1970s, post-1980s, and post-1990s, with the latter including those born from 1990 to 1999, consistent with the age range). The study period encompasses five survey waves: 2010, 2011, 2012, 2015, and 2017.

#### 3.2.4 Control variables

Control variables were selected based on theoretical relevance and data availability across all five survey waves. They can be categorized into three groups: (1) Basic Demographics: Gender (male = 1, female = 0) and household registration (hukou type, urban = 1, rural = 0). (2) Social Capital and Family Relations: Employment status (0 = unemployed, 1 = agricultural work, 2 = non-agricultural work), marital status (married = 1, else = 0), and number of children. (3) Socioeconomic Status (SES): Educational attainment (an ordinal variable from 1 = illiterate to 6 = postgraduate), subjective social status, and individual annual income (in logarithmic form). Detailed descriptions, coding, and descriptive statistics for all variables are presented in Table 1.

### 3.3 Statistical analyses

This study employs the Age, Period, and Cohort (APC) model for analyzing trends in research evolution. Given the multicollinearity among age, period, and generation factors, conventional linear regression models fail to address the identification challenges inherent in APC models. In response to this, scholars have progressively developed various methods to address the collinearity issue. Currently, the most representative and widely accepted approach is the Hierarchical APC-Cross-Classified Random Effects Model (HAPC-CCREM). It stands as one of the most recognized and extensively applied methods and has gained substantial academic acceptance and application in fields such as social sciences and epidemiology (40). The core strength of this model lies in its ability to effectively disentangle age, period, and cohort patterns through hierarchical Bayesian or restricted maximum likelihood estimation, all without imposing strong assumptions on the data.

This method establishes age as a primary-level variable, analyzing it as an individual (lifecycle) factor using fixed effects. Period and cohort variables are placed in the secondary layer, with each individual embedded within groups formed by period-cohort cross-classifications to break down linear relationships between these dimensions (41). In this framework, specific years and generation groups can be viewed as macro-level random factors influencing social status attainment, estimated through higher-order random effects. The hierarchical models nested structure transforms temporal dimensions at higher levels into environmental variables, whose associations on individuals manifest through adjustments to regression coefficients and intercepts. This approach effectively resolves collinearity issues inherent in original APC model variables while maintaining analytical rigor (42).

All models were estimated using restricted maximum likelihood (REML) with the lme4 package in R. Given the multi-stage stratified sampling design of the CGSS, individual-level survey weights (provided by the CGSS) were applied to ensure national representativeness. Continuous variables (age, log income) were grand-mean centered before entering the model to facilitate interpretation of random effects. Standard model diagnostic checks (e.g., residual plots, variance inflation factors) were performed and did not indicate any serious violations of model assumptions. To assess the stability of the cohort random slope estimates, we conducted two sensitivity analyses. First, we re-estimated the model using alternative cohort definitions (5-year intervals instead of decadal cohorts); the direction and significance of the cohort-level random slope variance remained consistent. Second, we re-ran the models without survey weights; the coefficient estimates and their significance were substantively unchanged. These checks support the reliability of the observed cohort heterogeneity reported in the Results.

The benchmark model is as follows:

First-level model (individual level):


$$\begin{array}{l} Y_{\mathrm{ijk}}=\beta_{0\mathrm{jk}}+\beta_{1\mathrm{jk}}PA_{\mathrm{ijk}}+\beta_{2\mathrm{jk}}\mathrm{AG}E_{\mathrm{ijk}}+\beta_{3\mathrm{jk}}\mathrm{AG}E_{\mathrm{ijk}}^{2}\\ +\beta_{4\mathrm{jk}}\mathrm{CO}N_{\mathrm{ijk}}+\varepsilon_{\mathrm{ijk}} \end{array}$$ (1)

where $Y_{\mathrm{ijk}}$ indicates the depressive symptoms level of individual i belonging to cohort j and period k ; $PA_{\mathrm{ijk}}$ indicates physical exercise status; $\mathrm{AG}E_{\mathrm{ijk}}$ indicates age; $\mathrm{AG}E_{\mathrm{ijk}}^{2}$ represents the square of age; $\mathrm{CO}N_{\mathrm{ijk}}$ represents control variables such as gender, education, household registration, number of children, and work status.

Second-level model (period and cohort level):


$$\beta_{0\mathrm{jk}}=\pi_{0}+\mu_{0j}+\nu_{0k}$$ (2)

The intercept $\beta_{0\mathrm{jk}}$ represents the mean depressive symptoms level of all individuals in cohort j and period k . $\mu_{0j}$ represents the random effect of cohort j on the intercept, and $\nu_{0k}$ represents the random effect of period k on the intercept, with $\mu_{0j}∼N(0,\sigma_{0\mu })$ and $\nu_{0k}∼N(0,\sigma_{0\nu })$ , both normally distributed.

Substituting Equation 2 into Equation 1 yields the full benchmark model:


$$\begin{array}{l} Y_{\mathrm{ijk}}=(\pi_{0}+\mu_{0j}+\nu_{0k})+\beta_{1\mathrm{jk}}PA_{\mathrm{ijk}}+\beta_{2\mathrm{jk}}\mathrm{AG}E_{\mathrm{ijk}}\\ +\beta_{3\mathrm{jk}}\mathrm{AG}E_{\mathrm{ijk}}^{2}+\beta_{4\mathrm{jk}}\mathrm{CO}N_{\mathrm{ijk}}+\varepsilon_{\mathrm{ijk}} \end{array}$$ (3)

Here, $\beta_{1\mathrm{jk}}$ represents the coefficient for the association between active physical exercise and depressive symptoms, compared with non-exercising individuals, after controlling for age, period, and cohort variables.

To examine the age pattern of the association between physical exercise and depressive symptoms, interaction terms between physical exercise and age (both linear and quadratic) were added at the first level. Substituting into model (3) yields the full model for the age pattern:


$$\begin{array}{l} \text{Yijk}=(\pi 0+\mu 0j+\nu 0k)+\beta 1\text{jkPAijk}+\beta 2\text{jkAGEijk}\\ +\beta 3\text{jkAGEijk}2+\beta 4\text{jkAGEijk}*\text{PAijk}\\ +\beta 5\text{jkAGEijk}2*\text{PAijk}+\beta 6\text{jkCONijk}+\text{εijk} \end{array}$$ (4)

To examine the generational pattern of physical exercise status, following the practice of Liu Yunbo et al., a group of models was added at the second level:


β1jk=γ1+u1j+v1k (5)

Here, $\gamma_{1}$ represents the average association between physical exercise and depressive symptoms after controlling for cohort differences; $u_{1j}$ represents the random effect of the j -th cohort on the relationship between physical exercise and depressive symptoms, with $u_{1j}∼N(0,\sigma_{1\mu })$ ; $v_{1k}$ represents the random effect of the k -th period on the relationship between physical exercise and depressive symptoms, with $v_{1k}∼N(0,\sigma_{1\nu })$ . If there is significant variation in the exercise-depressive symptoms association across cohorts or periods, the variance significance test for $\sigma_{1\mu }$ or $\sigma_{1\nu }$ will be significant.

Substituting Equation 5 into Equation 3 yields the full model for the generational pattern:


$$\begin{array}{l} \text{Yijk}=(\pi 0+\mu \;0\;j+\nu \;0\;k)+(\gamma 1+u1j+v1k)\;\text{PAijk}\\ +\beta \;2\;\text{jkAGEijk}+\beta \;3\;\text{jkAGEijk}2+\beta \;4\;\text{jkCONijk}+\varepsilon \;\mathrm{ijk} \end{array}$$ (6)

Based on the estimation results of the variance of the random effect for $u_{1j}$ (whether it is significant), we can determine whether there is a generational pattern in the association between good exercise habits (compared with those without good exercise habits) and depressive symptoms—that is, whether there is cohort heterogeneity in this association.

## 4 Results

### 4.1 Age heterogeneity: a non-linear age pattern suggestive of a U-shape

The final analytic sample consisted of 28,618 respondents aged 17–96 years (M = 44.38, SD = 15.19), with 48.1% male and 46.0% holding urban household registration (hukou). Recall that higher scores on our depressive symptom measure indicate less frequent depressive feelings (i.e., better mental health). To test Hypothesis 1 (H1), which posits a U-shaped pattern in the depressive symptoms advantage associated with regular physical exercise across the life course, Model (2) in Table 2 incorporates interaction terms between physical exercise and age (both linear and quadratic). The results provide strong support for this hypothesis. The coefficient for the interaction term between physical exercise and age is negative and statistically significant (*β* = −0.007, *p* < 0.001), while the coefficient for the interaction term with age squared is positive and significant (*β* = 0.000, *p* < 0.001). This combination of signs (negative linear × exercise, positive quadratic × exercise) is consistent with a non-linear age moderation pattern that suggests a U-shaped trajectory. The observed pattern is therefore interpreted as suggestive of a U-shaped association between exercise and depressive symptoms across the life course.

**Table 2:** APC model of depressive symptoms change trend.

Model	(1)	(2)	(3)	(4)
Fixed effects				
Physical exercise	0.113*** (0.004)	0.099*** (0.004)	0.092* (0.018)	0.071** (0.004)
age	−0.005*** (0.0003)	−0.008*** (0.0003)	−0.007*** (0.0003)	−0.007*** (0.0003)
age2	0.001*** (0.0001)	0.001*** (0.0001)	0.001*** (0.0001)	0.001*** (0.0001)
Exercise * Age (reference group: never exercised)				
Age of physical exercise		−0.007*** (0.0002)		
Age of physical exercise2		0.000*** (0.0001)		
Educational level	0.069*** (0.004)	0.071*** (0.004)	0.071*** (0.004)	0.069*** (0.004)
Sex	0.073*** (0.003)	0.078*** (0.003)	0.077*** (0.003)	0.079*** (0.003)
Marital status	0.222*** (0.006)	0.204*** (0.006)	0.210*** (0.006)	0.201*** (0.006)
Hukou	0.038** (0.012)	0.031* (0.013)	0.032* (0.013)	0.020 (0.018)
Log of income	0.013*** (0.003)	0.011*** (0.003)	0.011*** (0.003)	0.010*** (0.003)
Quantity of children	−0.037*** (0.003)	−0.035*** (0.003)	−0.034*** (0.003)	−0.032*** (0.003)
Working condition	0.011 (0.008)	0.021* (0.008)	0.022** (0.008)	0.027** (0.008)
Sports exercise * Household registration				0.052* (0.026)
Exercise * Household registration * Age				0.007*** (0.0003)
Reference group: Eastern region				
middle part	−0.063*** (0.008)	−0.063*** (0.008)	−0.063*** (0.008)	−0.064*** (0.008)
the west area	−0.127*** (0.008)	−0.126*** (0.008)	−0.126*** (0.008)	−0.126*** (0.008)
Second-order random effect variance				
Generations (reference group: never exercised)				
Physical exercise			0.002** (0.005)	0.001** (0.006)
The intercept term	0.001*** (0.0003)	0.001*** (0.0003)	0.013*** (0.0003)	0.002*** (0.0003)
Period (reference group: never exercised)				
Physical exercise			0.001 (0.0003)	0.001 (0.0003)
The intercept term			0.001*** (0.0003)	0.001*** (0.0003)
N	28,618	28,618	28,618	28,618
Fitting BIC	78,590	78,544	78,533	78,562

*p < 0.05, **p < 0.01, ***p < 0.001. Standard errors (SE) are shown in parentheses.

**Figure 1:**
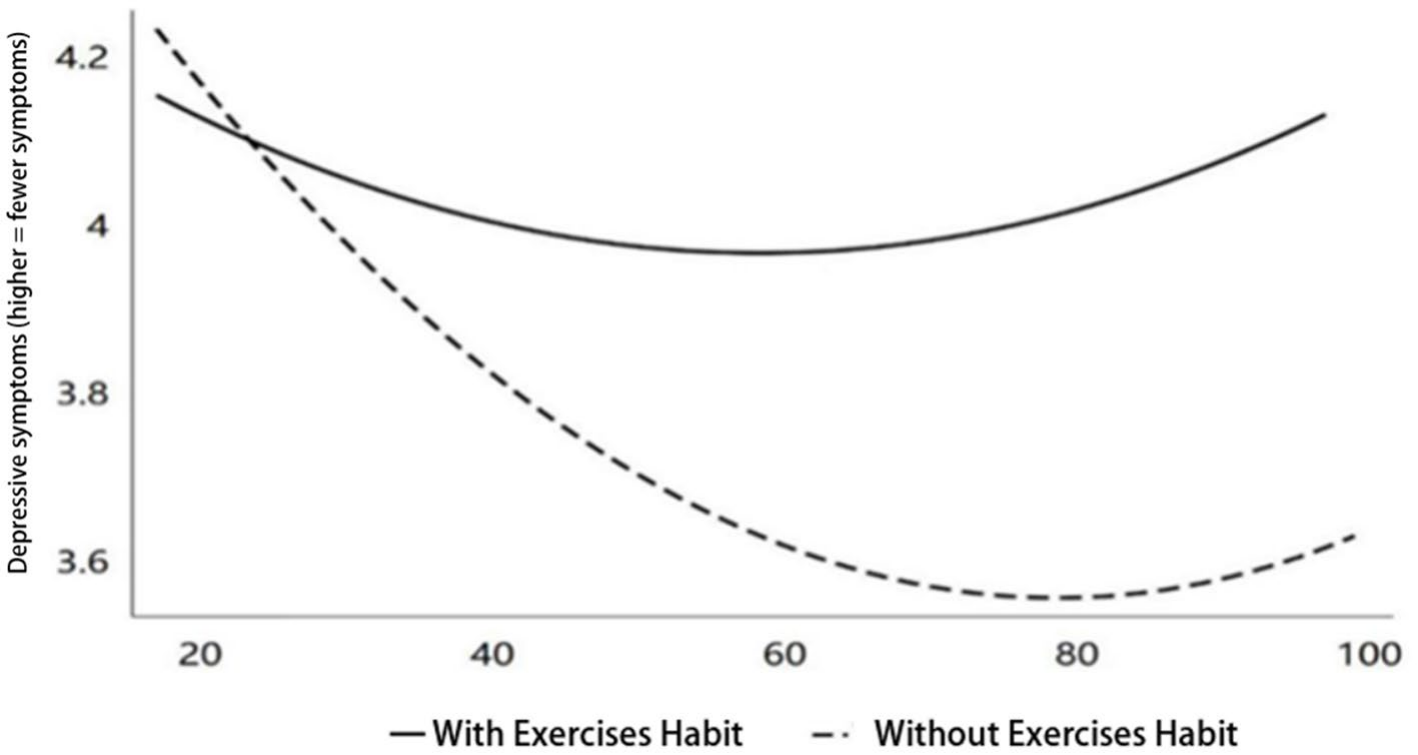
Age pattern of depressive symptoms by exercise habit.

As visually depicted in Figure 1, the positive association with regular exercise is not constant but evolves dynamically with age. This positive association is most pronounced during young adulthood and later life, forming the two elevated ends of the “U.” During the middle-age period (approximately 30–55 years), the depressive symptoms advantage of exercisers over non-exercisers attenuates, though it remains positive. This pattern suggests that while regular exercise is consistently positively associated with depressive symptoms, its relative pattern of association is challenged during mid-life, a period typically characterized by peak career and family responsibilities (“role overload”). Nonetheless, maintaining an exercise habit is associated with a less steep decline in depressive symptoms during mid-life compared to those who are inactive. These findings provide preliminary evidence supporting a non-linear, possibly U-shaped age pattern, thus offering preliminary support for H1.

### 4.2 Cohort heterogeneity: decline and resurgence across generations in the exercise-depressive symptoms association

Hypothesis 2 (H2) predicted significant variation in the positive association between exercise and depressive symptoms across birth cohorts, following a pattern of decline and subsequent recovery. This is tested in Model (3) by allowing the slope of the physical exercise variable to vary randomly across cohorts. The results show a significant variance component for the cohort-level random effect on the exercise slope (σ^2^ = 0.002, *p* < 0.01), indicating substantial heterogeneity across generations. In contrast, the period-level random effect was non-significant, confirming that the differences are generational rather than driven by specific survey years.

**Figure 2:**
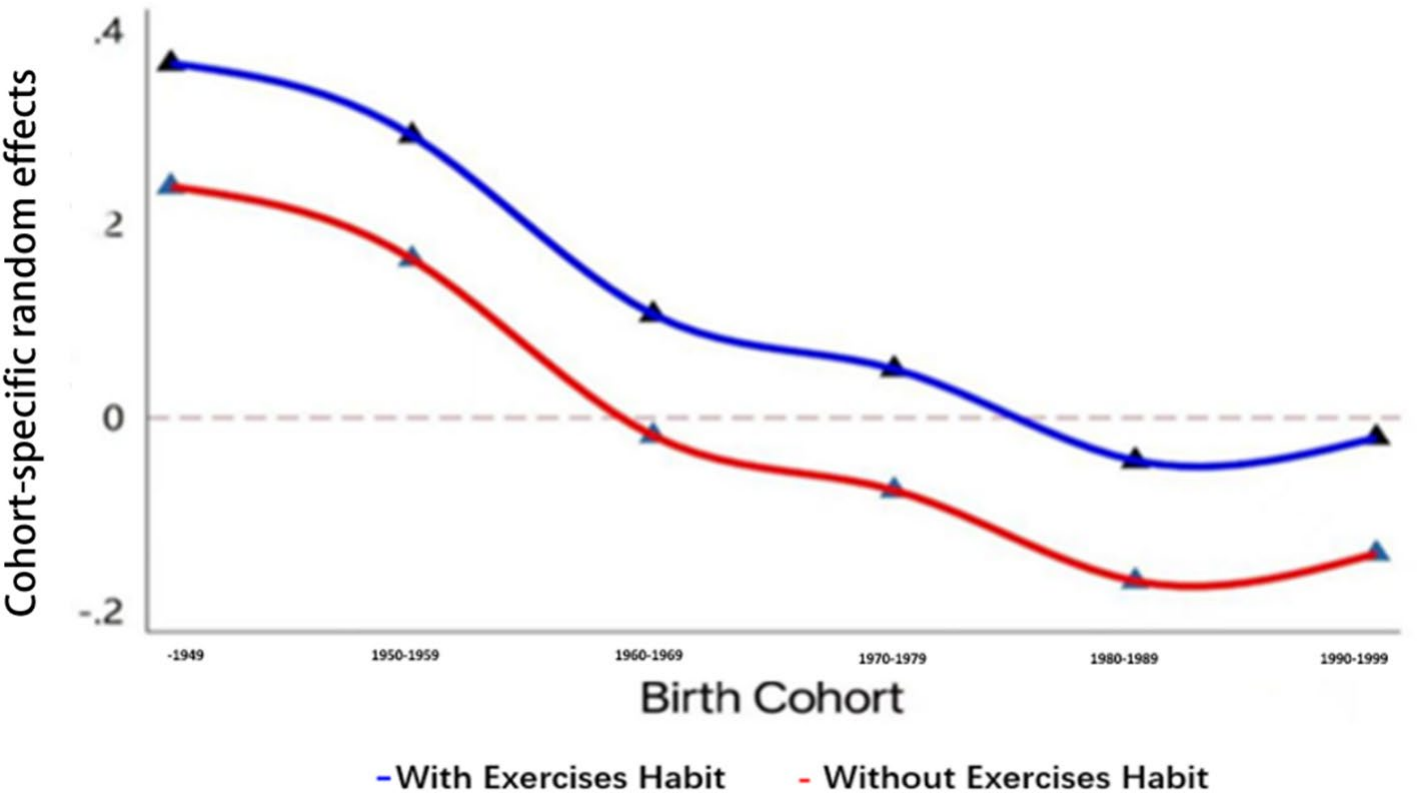
Cohort-specific deviations in depressive symptoms by exercise habit.

Figure 2 displays cohort-specific deviations estimated from the random slope component of the APC model. Because the original depressive symptom score ranges from 1 (always depressed) to 5 (never depressed), higher scores indicate fewer depressive symptoms. The figure presents deviations from the overall predicted level: positive deviations indicate worse depressive symptoms (i.e., lower original scores) than average, whereas negative deviations indicate better depressive symptoms (i.e., higher original scores) than average. As shown, the cohort-specific deviations highlight how the effect of exercise on depressive symptoms varies across birth cohorts. The earliest cohort (pre-1949) exhibits the largest gap, indicating a strong positive association between exercise and depressive symptoms (difference = 0.22 in predicted score). This gap narrows substantially for cohorts born in the 1950s through the 1980s, coinciding with periods of intense economic reform and material pursuit where exercise may have been perceived as a lower-priority “luxury.” However, a notable widening of the gap is observed for the post-1990s cohort. This resurgence suggests a shift in the function of exercise for younger generations—from a discretionary enhancer to a necessary tool for managing modern psychosocial stresses in an era leaning towards post-materialist values. Thus, the empirical evidence aligns with the theorized pattern of decline and recovery, supporting H2.

### 4.3 Moderating role of urban–rural disparities

Hypothesis 3 (H3) concerned the moderating role of the urban–rural divide. Model (4) introduces interaction terms between physical exercise and household registration (hukou) status. The coefficient for the interaction term Physical Exercise * Urban Hukou is positive and significant (*β* = 0.052, *p* < 0.05). This indicates that the positive association between regular exercise and depressive symptoms is significantly stronger for urban residents compared to rural residents.

**Figure 3:**
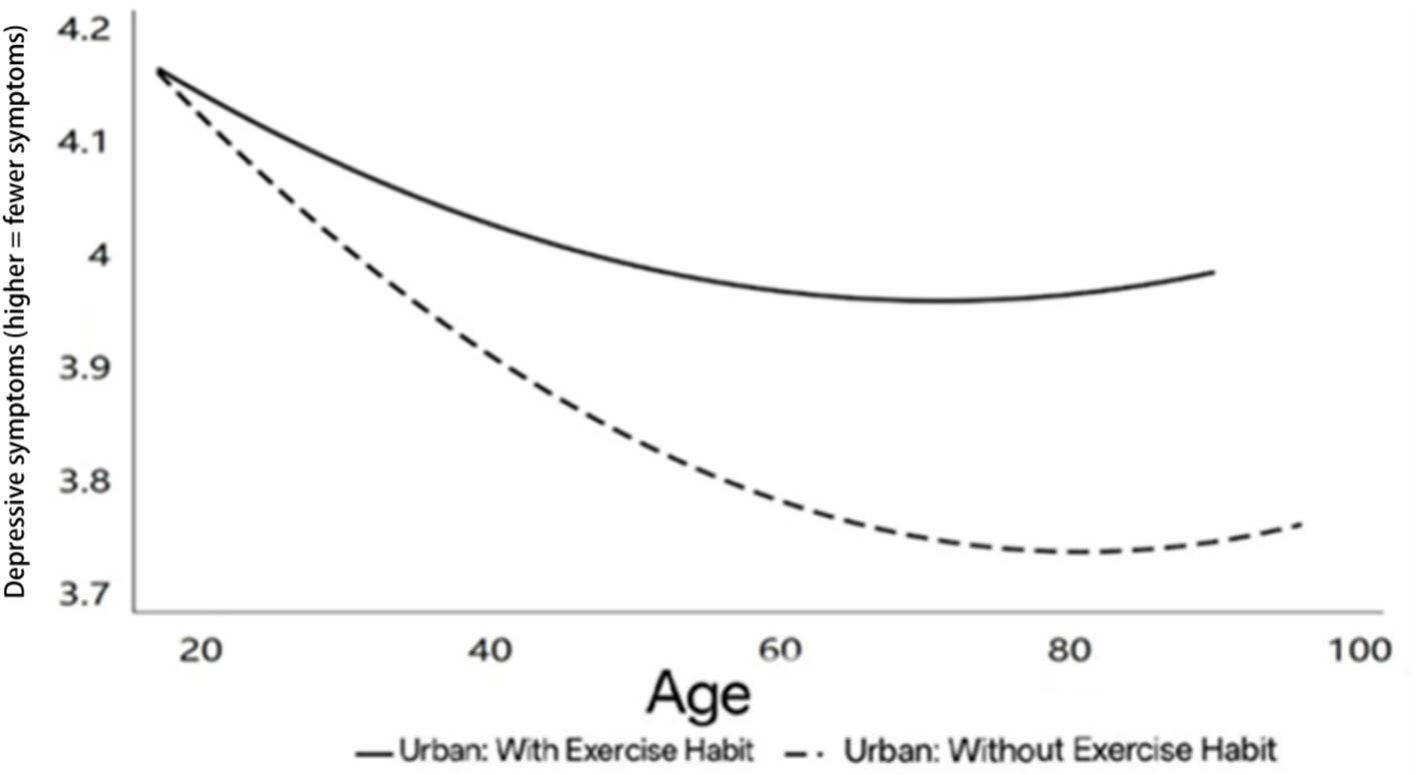
Age pattern of depressive symptoms by exercise habit – urban residents.

**Figure 4:**
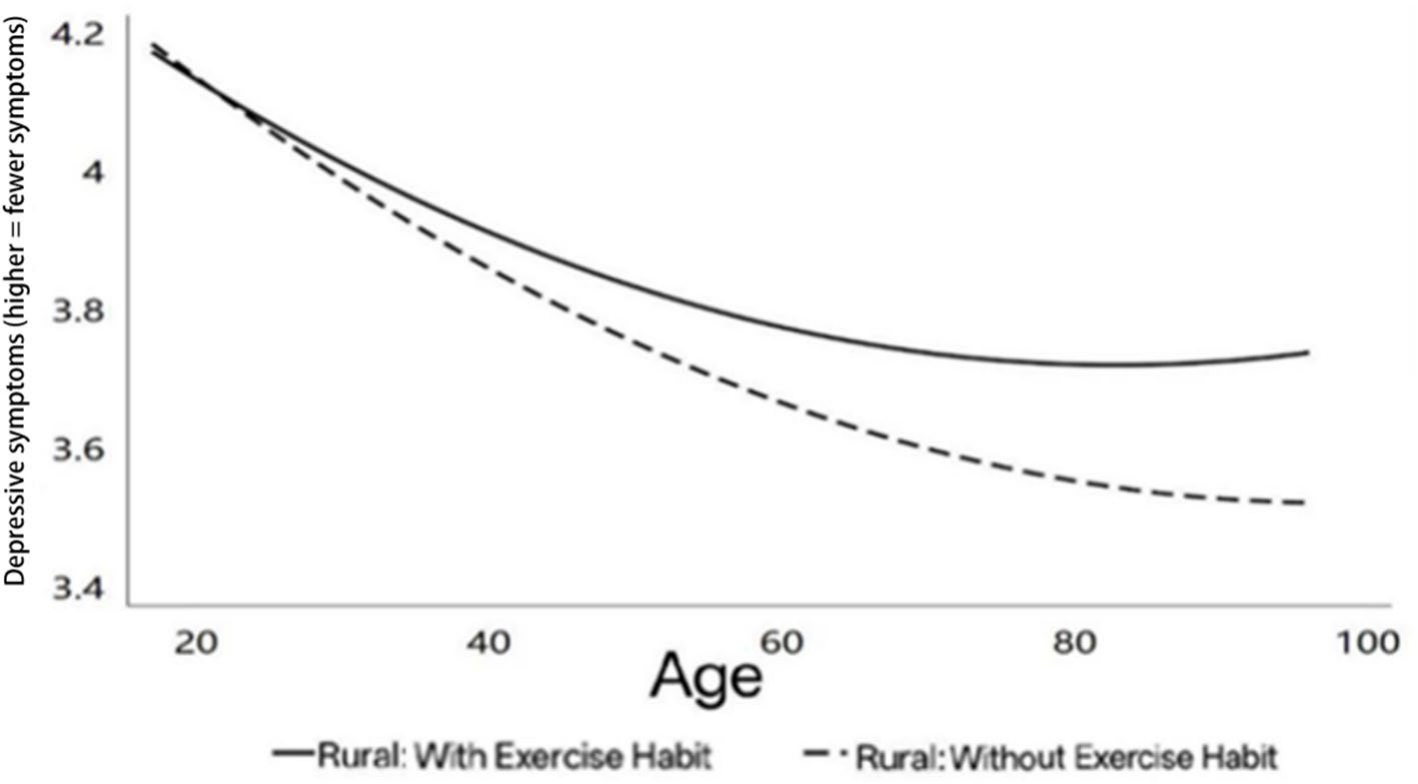
Age pattern of depressive symptoms by exercise habit – rural residents.

The age-specific dynamics of this moderation are further illustrated in Figures 3, 4. Urban exercisers (Figure 3) show a higher and more stable trajectory of depressive symptoms across the lifespan, with the exercise-related advantage being particularly evident during the middle-age years (a period often associated with high stress). In contrast, while rural exercisers (Figure 4) also show a positive association relative to their non-exercising rural counterparts, the absolute level of depressive symptoms and the magnitude of this association are consistently lower. The gap is most pronounced during early and middle adulthood. This observation is consistent with the theoretical argument that urban environments, with their superior infrastructure and richer social contexts for exercise, may be associated with stronger positive psychosocial correlates of exercise, whereas structural constraints in rural areas may be associated with a more limited positive association. However, as we directly used hukou as a proxy, these mechanisms remain inferential and require direct testing with measures of actual living environment and resource access. Thus, H3 is supported.

### 4.4 Robustness checks

To assess the stability of our findings, we conducted three sensitivity analyses. First, we re-estimated the main model using alternative cohort definitions (5-year intervals instead of decadal cohorts). Second, we re-ran the models without survey weights (i.e., deliberately omitting the CGSS sampling weights) and also omitted the period random effect. Third, to address concerns that classifying monthly exercise as “regular” might lead to an overestimation of meaningful exercise habits, we re-estimated the main model using a stricter definition: daily or weekly exercise was coded as regular (1), while monthly or less frequent exercise was coded as non-regular (0). Thus, monthly exercise was recoded as non-regular (not excluded from the sample). The results are presented as Sensitivity 3 in Table 3. As shown in the table, all key patterns remained statistically significant, and their effect sizes were similar to those in the main model, confirming the robustness of our conclusions.

**Table 3:** Sensitivity analyses: alternative cohort definition (5-year intervals) and without survey weights.

Variable	Main model (Table 2, Model 4)	Sensitivity 1 (5-year cohorts)	Sensitivity 2 (without weights)	Sensitivity 3 (daily/weekly only)
Fixed effects				
Physical Exercise (PE)	0.071** (0.004)	0.069** (0.005)	0.073** (0.004)	0.063** (0.007)
Age	−0.007*** (0.0003)	−0.007*** (0.0003)	−0.007*** (0.0003)	−0.006*** (0.0003)
Age^2^	0.001*** (0.0001)	0.001*** (0.0001)	0.001*** (0.0001)	0.001*** (0.0001)
Age × PE	−0.007*** (0.0002)	−0.006*** (0.0002)	−0.007*** (0.0002)	−0.006*** (0.0002)
Age^2^ × PE	0.001*** (0.0001)	0.001*** (0.0001)	0.001*** (0.0001)	0.001*** (0.0001)
PE × Urban Hukou	0.052* (0.026)	0.048* (0.030)	0.050* (0.028)	0.048* (0.031)
Random effect variance				
Cohort: PE slope (σ^2^)	0.001** (0.006)	0.001** (0.007)	0.001** (0.006)	0.001** (0.008)
*N*	28,618	28,618	28,618	28,618

*p < 0.05, **p < 0.01, ***p < 0.001. Standard errors in parentheses. Sensitivity 1 used 5-year birth cohorts (instead of decadal cohorts). Sensitivity 2 omitted survey weights (i.e., the main model was re-estimated without applying weights) to assess the influence of the sampling design; the period random effect was also omitted in this specification. Sensitivity 3 used a stricter definition of regular exercise (daily or weekly only, recoding monthly exercise as non-regular). All models used the same analytic sample as the main model.

## 5 Discussion

The findings of this study reveal that the positive association between physical exercise and depressive symptoms is not uniform but is dynamically shaped by age, cohort, and structural context. In what follows, we first report the empirical patterns directly revealed by our HAPC model (age, cohort, and hukou heterogeneity). We then offer theoretical interpretations of these patterns, drawing on existing sociological and psychological theories. It should be noted that these explanations are merely speculative and require direct verification in future research.

First, the positive association between physical exercise and depressive symptoms exhibits a non-linear age pattern suggestive of a U-shape, being most pronounced in young adulthood and old age, while weakening somewhat in middle age but still showing a positive association. This reflects the influence of social role transitions across the life course on the significance and perceived efficacy of health behaviors. This finding is highly consistent with life course theory and social role theory (43, 44). During adolescence, individuals are in the stage of forming social identities and launching their careers. Physical exercise serves not only as a release valve for physiological stress but also as a crucial social ritual for building social capital and integrating into peer groups. Its functions of social integration and stress reduction are prominent, showing the strongest positive psychological correlates (45, 46). Upon entering middle age, individuals face “role overload” from multiple responsibilities such as career development and family care, resulting in a severe squeeze on personal discretionary time (47). Although the physiological advantages of physical exercise persist, its relative priority as a leisure and psychological enrichment activity declines, leading to a weakening of the positive association between exercise and depressive symptoms compared to young adulthood and forming the trough of the U-shaped curve. Nevertheless, compared to those who do not exercise, consistent exercise can still significantly correlate with a slower rate of mental health decline caused by midlife stress (i.e., fewer depressive symptoms), demonstrating the long-term protective pattern of habit formation. In old age, as individuals withdraw from productive social roles, health maintenance becomes the central concern. Physical exercise (such as walking and tai chi) once again becomes central to daily life, and its role in maintaining physical function, promoting social interaction, and enhancing a sense of control and meaning in life becomes crucial, with positive psychological correlates rising once more (48). This suggests that public policy should adopt a life-course perspective, providing tailored physical exercise promotion programs that address the core life roles and psychological needs of different age groups.

Second, the positive association between physical exercise and depressive symptoms shows significant generational heterogeneity, following a trajectory of “decline followed by recovery.” The earliest generations showed the strongest positive association; this association declined among the middle cohorts (born 1950s-1980s) and showed a notable recovery among the post-1990s cohort. While these empirical patterns are robust, the explanations we offer below are speculative and should be interpreted as possible mechanisms rather than direct evidence. One possible interpretation is that the decline among middle cohorts coincided with China’s rapid economic reform and the rise of materialist values, where immediate economic pursuits may have crowded out leisure-time exercise as a low-priority activity (49). For the earliest cohorts (pre-1949 and 1950s-60s), those who maintained regular exercise were often from relatively privileged socioeconomic backgrounds, so exercise might have functioned as a “value-added” lifestyle enhancement (50). For the post 1990s cohort, growing up in greater material abundance, post materialist values (e.g., quality of life, self expression, mental health) may have begun to emerge (51). At the same time, they face unique modern pressures such as educational “involution,” career precarity, and digital-age anxieties (52). It is plausible that, in this context, physical exercise has shifted from a discretionary leisure activity to a strategic tool for stress management and resilience building (28). However, we did not directly measure materialist/post-materialist values, work pressure, digital anxiety, or changing meanings of exercise. Therefore, these interpretations remain speculative and should be tested in future studies with appropriate mediating variables. The observed cohort heterogeneity nevertheless provides a robust empirical pattern that invites further investigation into the social mechanisms linking macro-level transformations to individual health behaviors (53). We also acknowledge that classifying monthly exercise as ‘regular’ may overestimate meaningful exercise habits. Future studies using a stricter definition (e.g., daily or weekly exercise only) are needed to confirm our findings.

Finally, the urban–rural dichotomy (operationalized by hukou status) significantly moderates the association between physical exercise and depressive symptoms. However, hukou is a legal registration status and may not perfectly reflect an individual’s actual living environment, access to sports facilities, or community resources. We therefore interpret the moderating role of hukou as an indicator of structural inequality, while acknowledging that the mechanisms discussed below are inferred rather than directly tested. One plausible explanation for this moderation is rooted in structural inequalities in resource allocation and the social environment (54). Urban areas typically concentrate higher-quality public sports facilities, green spaces, and organized community activities, which may lower the objective barriers to regular exercise (55). At the level of social relations, physical exercise in urban settings may be more easily embedded in rich social contexts (56). Modern urban spaces such as gyms, sports clubs, and square dance communities can function as “incubators of social capital” (57). Regular participation may not only promote physical fitness but also serve as a ritual of social interaction, providing sustained emotional support, reinforcing social identity, and fostering a sense of belonging. Through these psychosocial pathways, it could amplify the positive psychological correlates of physical exercise (58). In contrast, rural residents often face structural constraints such as relative economic pressure and weaker social security (59), and may lack the social sports environments necessary to cultivate such social capital. Their physical activities may be more isolated and sporadic, which could weaken the positive associations with depressive symptoms generated through social capital pathways. Critically, our findings reveal that both the depressive symptoms levels of exercisers and the magnitude of the positive association with exercise are significantly lower among rural residents compared to their urban counterparts, with the most pronounced gaps occurring during youth and middle age. These patterns are consistent with the hypothesis that structural disadvantages constrain the mental health benefits of exercise (i.e., the association with fewer depressive symptoms), but we did not directly measure access to facilities, social capital, or built environment characteristics. Future studies should incorporate such direct measures to test the proposed mechanisms.

This study is subject to several limitations that point to valuable avenues for future research. First and most significantly, the reliance on repeated cross-sectional data, while analyzed using an APC model to infer population-level trends, precludes firm causal inference about the exercise-depressive symptoms relationship at the individual level. It cannot delineate whether changes in exercise are associated with changes in depressive symptoms, or vice versa, or account for unobserved time-invariant individual characteristics. Future longitudinal studies with panel data are essential to establish within-person causality and to explore these potential bidirectional dynamics over time. Second, the measurement of physical exercise was based solely on self-reported frequency (“Do you engage in physical exercise in your spare time?” dichotomized as regular vs. irregular). This single-dimensional measure lacks information on exercise intensity, duration, type (e.g., aerobic vs. team sports), or social context, all of which may differentially correlate with depressive symptoms. The CGSS data used in this study did not collect such detailed information, preventing us from conducting analyses on intensity or duration. Future research should employ more nuanced and objective measures (e.g., accelerometers, detailed questionnaires capturing intensity, duration, and type) to disentangle these specific dose–response associations. Third, our measure of the outcome variable was limited to a single item assessing the frequency of depressive feelings over the past four weeks. This item captures only the depression/distress dimension of mental health, whereas mental health is a broader construct encompassing emotional well-being, psychological functioning, anxiety, stress, and social aspects. Therefore, our findings should be interpreted specifically in relation to depressive symptoms rather than mental health as a whole. Future studies should employ validated multidimensional scales (e.g., PHQ-9, GAD-7, WHO-5 Well-Being Index, or SF-12 Mental Component Summary) to capture the full spectrum of mental health outcomes and to examine whether the observed patterns extend to other dimensions. Fourth, while the theoretical framework posits pathways such as social capital accumulation and stress reduction, the empirical model did not include relevant mediating variables for direct testing. Consequently, the analysis could not thoroughly dissect the specific mechanisms through which the positive association of exercise with depressive symptoms is generated or why this association varies across groups. Future work should explicitly incorporate and test these hypothesized mediators (e.g., social network size, perceived stress, self-efficacy) to illuminate the black box between behavior and psychological outcome. Finally, the operationalization of birth cohorts into decadal intervals, though standard, may mask more nuanced historical influences. Grouping cohorts aligned with major socio-historical events (e.g., pre- and post-economic reform in China, the advent of the internet) could provide a more accurate and theoretically grounded capture of the impact of specific social changes on the meaning and correlates of physical exercise with depressive symptoms. Sixth, while the HAPC-CCREM is a widely used tool for disentangling age, period, and cohort patterns, it does not completely eliminate the identification problem. Its validity depends on the number of periods and cohorts and on the assumption that the random effects are uncorrelated with the predictors. In this study, we used only five survey waves (2010–2017) and seven decadal cohorts, which limits the precision of random effect estimation, particularly for cohort random slopes. Although our robustness checks using alternative cohort groupings (5-year intervals) yielded consistent patterns, the relatively small number of period and cohort units may constrain the generalizability of the cohort heterogeneity we observed. Future studies with longer time spans and more survey waves are needed to confirm our findings.

## 6 Conclusion

Using the HAPC model, this study systematically examined the age, period, and generational patterns of physical exercise habits in relation to depressive symptoms (as an indicator of mental health) and tested the moderating role of urban–rural differences. The main conclusions are as follows: (1) The advantage associated with physical exercise in terms of depressive symptoms exhibits a non-linear age pattern suggestive of a U-shape, being most pronounced in young adulthood and old age, and somewhat diminished in middle age but still positively associated. This reflects the impact of shifting social roles across the life course on the meaning and perceived efficacy of health behaviors. (2) The association between physical exercise and depressive symptoms exhibits clear generational heterogeneity, following a trajectory of “decline followed by recovery.” Earlier generations showed the strongest positive association; this association declined among middle cohorts and rebounded among the post-1990s cohort. These patterns may be tentatively interpreted in light of China’s socio-economic transformation and changing values, but the proposed mechanisms remain speculative and require direct testing in future research. (3) The urban–rural dichotomy (using hukou as a proxy for structural inequality) significantly moderates the association between physical exercise and depressive symptoms. Urban residents exhibit a stronger positive association and more pronounced stress-alleviating patterns; in contrast, rural residents show a relatively limited positive association. These patterns suggest possible differences in resource access and social contexts, but we did not directly measure these factors. Therefore, the proposed mechanisms should be interpreted with caution and tested in future research. Overall, the correlates of physical exercise with respect to depressive symptoms are not static but rather a dynamic pattern shaped by both the individual life course and macro-social changes. Against the backdrop of Chinese society’s transition from material accumulation to post-materialist values, the role of physical exercise may be undergoing a profound transformation. Therefore, future public mental health promotion strategies should abandon a “one-size-fits-all” approach and fully consider individuals’ life stages, generational experiences, and the urban–rural structural environments in which they live. By formulating and implementing more targeted, differentiated, and precise promotion strategies, these approaches can more effectively be associated with better mental health outcomes for the entire population and promote health equity. We emphasize that while our empirical model robustly demonstrates heterogeneity by age, cohort, and hukou, the theoretical mechanisms we propose (e.g., social role transitions, materialist/post-materialist values, social capital) are not directly tested and should be interpreted with caution.

## Data Availability

The original contributions presented in the study are included in the article/supplementary material, further inquiries can be directed to the corresponding author.
